# Unlocking the mysteries of VLDL: exploring its production, intracellular trafficking, and metabolism as therapeutic targets

**DOI:** 10.1186/s12944-023-01993-y

**Published:** 2024-01-12

**Authors:** Jingfei Chen, Zhenfei Fang, Qin Luo, Xiao Wang, Mohamad Warda, Avash Das, Federico Oldoni, Fei Luo

**Affiliations:** 1 Research Institute of Blood Lipid and Atherosclerosis, the Second Xiangya Hospital, Central South University, Changsha, Hunan 410011 China; 2 Reproductive Medicine Center, Department of Obstetrics and Gynecology, Research Institute of Blood Lipid and Atherosclerosis, The Second Xiangya Hospital, Central South University, Changsha, Hunan 410011 China; 3 Department of Cardiovascular Medicine, The Second Xiangya Hospital, Central South University, Changsha, Hunan 410011 China; 4 State Key Laboratory of Membrane Biology, Peking University, Beijing, 100871 China; 5 Department of Biochemistry and Molecular Biology, Faculty of Veterinary Medicine, Cairo University, Giza, 12211 Egypt; 6 Department of Physiology, Faculty of Veterinary Medicine, Ataturk University, Erzurum, 25240 Turkey; 7 Department of Pathology, Beth Israel Deaconess Medical Center, Harvard Medical School, Boston, MA 02215-5400 USA; 8 Department of Molecular Genetics, University of Texas Southwestern Medical Center, Dallas, TX USA

**Keywords:** Atherosclerotic cardiovascular disease, Very low-density lipoprotein, Low-density lipoprotein, LDL receptor-independent pathway

## Abstract

Reducing circulating lipid levels is the centerpiece of strategies for preventing and treating atherosclerotic cardiovascular disease (ASCVD). Despite many available lipid-lowering medications, a substantial residual cardiovascular risk remains. Current clinical guidelines focus on plasma levels of low-density lipoprotein (LDL). Recent attention has been given to very low-density lipoprotein (VLDL), the precursor to LDL, and its role in the development of coronary atherosclerosis. Preclinical investigations have revealed that interventions targeting VLDL production or promoting VLDL metabolism, independent of the LDL receptor, can potentially decrease cholesterol levels and provide therapeutic benefits. Currently, methods, such as mipomersen, lomitapide, and ANGPTL3 inhibitors, are used to reduce plasma cholesterol and triglyceride levels by regulating the lipidation, secretion, and metabolism of VLDL. Targeting VLDL represents an avenue for new lipid-lowering strategies. Interventions aimed at reducing VLDL production or enhancing VLDL metabolism, independent of the LDL receptor, hold promise for lowering cholesterol levels and providing therapeutic benefits beyond LDL in the management of ASCVD.

## Introduction

Despite significant therapeutic advances, atherosclerotic cardiovascular disease (ASCVD) remains one of the major causes of mortality worldwide. High cholesterol and high triglyceride (TG) levels are considered important risk factors for the development of ASCVD [1]. Triglyceride-rich lipoproteins (TRLs) are a type of lipoprotein that are rich in triglycerides [2]. It consists mainly of very low-density lipoprotein (VLDL) produced by the liver and chylomicrons generated in the intestines. The TRLs play a vital role in lipid metabolism by transporting triglycerides and lipids to peripheral tissues for energy production or storage. VLDL facilitates fatty acid supply during fasting to muscles, and chylomicrons primarily provide fatty acids to adipose tissue. This regulation is predominantly governed by ANGPTL4/3/8 [3]. TRLs are converted into smaller and denser particles called TRL remnants through the action of lipoprotein lipase and other enzymes. These TRL remnants have fewer TGs and phospholipids but are enriched in cholesteryl esters. Elevated levels of TGs are linked to a higher risk of ASCVD. Unfortunately, the existing therapies aimed at lowering TGs have shown limited effectiveness in reducing cardiovascular risk [4]. TRLs have been identified as a causal risk factor for ASCVD [5, 6] and reported to be more atherogenic per particle than LDL cholesterol (LDL-C) [7, 8]. Moreover, elevated plasma TRL levels are dose-dependently associated with acute pancreatitis risk. In patients with persistently high triglyceride levels despite high-intensity statin therapy, guidelines often suggest considering adjunctive treatments such as fibrates, niacin, or long-chain omega-3 fatty acids [9]. Diet and exercise play crucial roles in reducing TRLs, with clinical guidelines emphasizing them as foundational for TRL reduction [5]. Recent studies have shown that VLDL-C plays a role in the etiology and progression of ASCVD. It has been estimated that the level of VLDL-C may account for 46% of the risk of apolipoprotein B (APOB)-related myocardial infarction [10, 11]. As a major source of TRL, especially during the fasting state, VLDL is a critical player in lipid metabolism. However, therapeutic strategies safely targeting the secretion or clearance of VLDL are lacking. A better understanding of the mechanism of ASCVD related to circulating VLDL can help identify new therapeutic targets for patients with a residual risk of ASCVD. Here, we summarize the latest research progress on VLDL metabolism and its potential as a lipid-lowering target, hoping to provide a reference for future research.

## Composition of VLDL

**Fig. 1:**
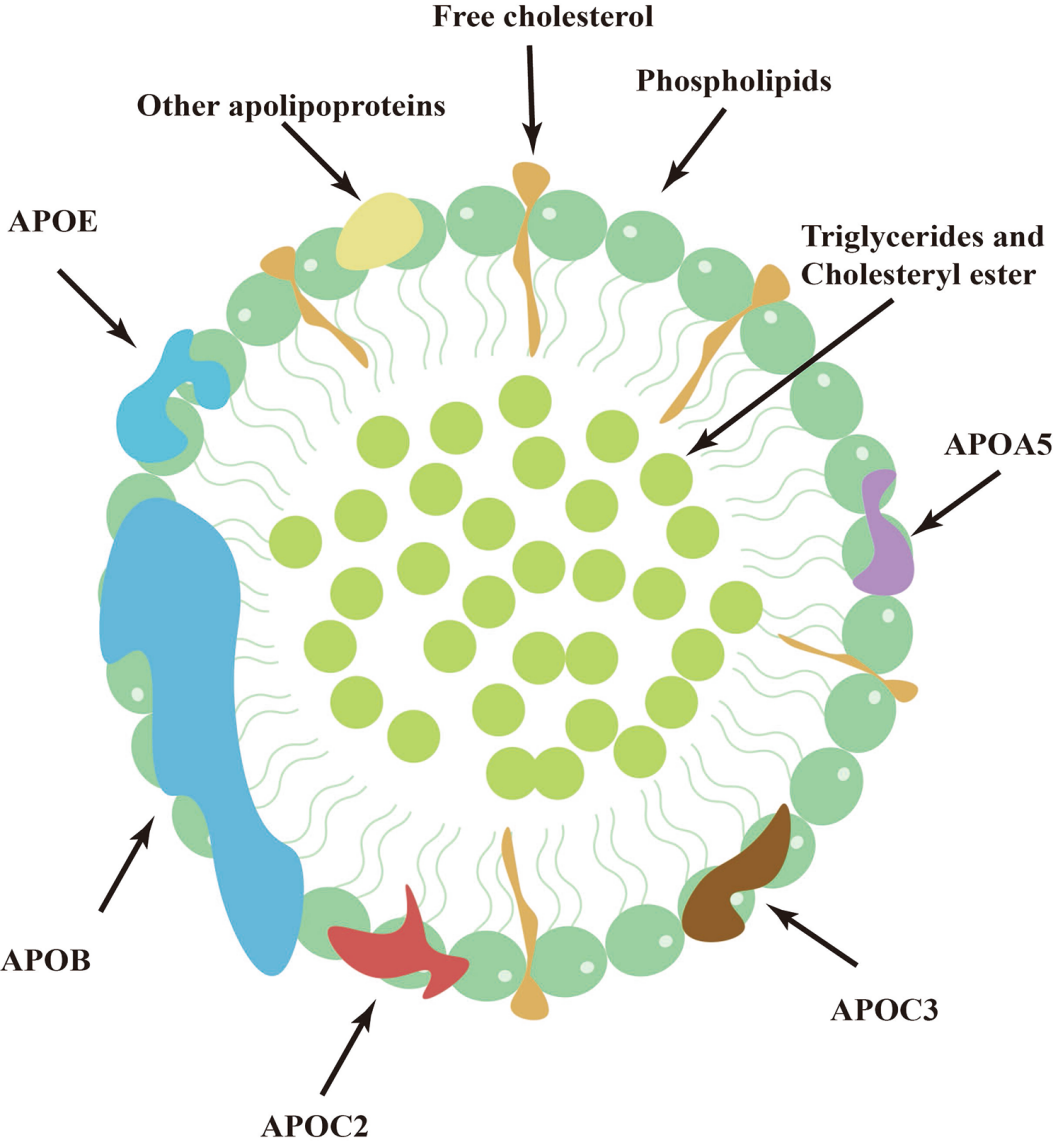
Schematic figure of apolipoproteins and the lipid composition of VLDL. Very low-density lipoprotein (VLDL) contains apolipoprotein B100 (APOB) on its surface as a structural protein and a surface monolayer of phospholipids; free cholesterol; and apolipoproteins such as apolipoprotein C2 (APOC2), APOC3, apolipoprotein E (APOE), and APOA5. The central core of VLDL contains cholesterol esters and triglycerides

Chylomicrons, VLDL, intermediate-density lipoprotein (IDL; VLDL remnants), LDL, and high-density lipoprotein (HDL) are the main categories of lipoproteins that can range in density from low to high [12]. The density of VLDL is very low, ranging from 0.93 to 1.006 g/mL, and the size of the mass is approximately 30–80 nm. VLDL has APOB as its structural protein and contains other apolipoproteins, such as apolipoprotein C2 (APOC2), APOC3, apolipoprotein E (APOE), and apolipoprotein A5 (APOA5) [12] (Fig. 1). APOC3 is a smaller apolipoprotein consisting of 79 amino acid residues. It is predominantly located on TRLs and HDL in circulation [13]. APOC3 functions as a lipoprotein lipase (LPL) inhibitor, impeding the breakdown of TRLs [13]. Deficiency or inhibition of APOC3 can lead to decreased TG levels [13]. In addition to protein, VLDL primarily carries TG while also containing cholesterol esters and phospholipids [12]. However, the composition of VLDL particles is subjected to changes, and the amount of TG carried by each particle can also vary dramatically. Hydrophobic lipids (TG and cholesterol ester) form the core of VLDL. Phospholipids form a monolayer on the hydrophilic surface with unesterified cholesterol and exchangeable apolipoproteins.

## Biogenesis and lipidation of VLDL

**Fig. 2:**
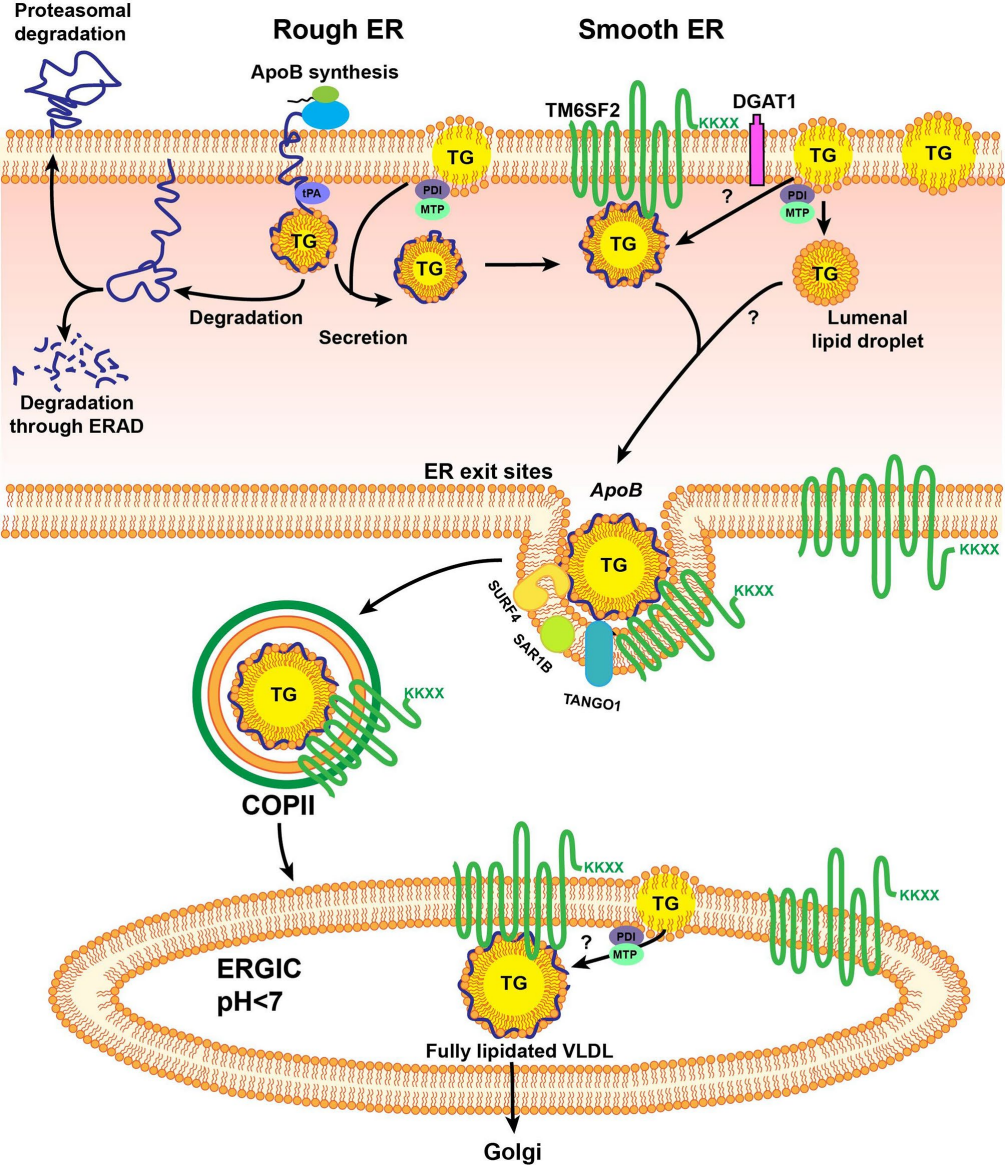
Biogenesis, lipidation and intracellular trafficking of VLDL. The assembly of VLDL in the liver begins with the cotranslational translation of apolipoprotein B (APOB) across the rough endoplasmic reticulum (RER) membrane. Microsomal triglyceride transfer protein (MTP) plays an essential role in the initial lipidation of APOB by extracting phospholipids and triglycerides (TGs) from the endoplasmic reticulum (ER). Without sufficient lipidation, nascent APOB is degraded. After the initial lipidation, VLDL is further lipidated with a large amount of lipids to form mature VLDL. To exit the endoplasmic reticulum, VLDL is packaged into transport vesicles called coat protein complex II (COPII), which is initiated by the activation of the small Ras-like GTPase SAR1.

The biosynthesis of VLDL is a multistep process that begins in the rough endoplasmic reticulum (RER) (Fig. 2). Hepatic VLDL assembly starts with the cotranslational translocation of APOB across the rough ER membrane, where microsomal triglyceride transfer protein (MTP) helps in the first lipid decoration of APOB [14]. Without sufficient lipidation, nascent APOB is degraded [15]. Thus, the biogenesis of VLDL relies on the availability of APOB, phospholipids and TGs. TGs include saturated and monoenoic fatty acids, while phospholipids constitute the most significant proportion of polyunsaturated fatty acids.

With the availability of lipids, partially lipidated APOB (pre-VLDL) will become fully lipidated with the bulk of lipids. The lipidation of very low-density lipoprotein (VLDL) is a two-step process [14, 16, 17]. The first step of lipidation occurs in the lumen of RERs during the translation of APOB, where a small amount of lipid combines with APOB to form pre-VLDL with the assistance of MTP [15]. The second step of VLDL lipidation involves pre-VLDL receiving a large number of lipids to form mature VLDLs. However, where this event occurs, only in the endoplasmic reticulum (ER) or in the Golgi apparatus, is controversial [18–20]. Previous studies have shown that transmembrane 6 superfamily member 2 (TM6SF2), a polytopic ER protein, plays a role in this second step of VLDL lipidation [16, 21]. The protein localizes to the smooth ER and the ER-Golgi intermediate compartment (ERGIC) [16, 21]. Researchers have proposed that two types of VLDL exist: triglyceride-rich VLDL1 and triglyceride-poor VLDL2 [22, 23]. Triglyceride-poor VLDL2 was formed after pre-VLDL acquired additional lipids [22, 23]. Triglyceride-poor VLDL2 can be directly secreted from hepatocytes or fused with APOB-free lipid droplets to form triglyceride-rich VLDL1. Other proteins are also reported to be involved in the assembly of VLDL, including cell death-inducing DFF45-like effector B (CIDEB), phospholipid transfer protein (PLTP) [24, 25], lysophosphatidylcholine acyltransferase 3 (LPCAT3) [26] and transmembrane protein 41B (TMEM41B) [27]. The most recent study reported that tissue-type plasminogen activator (tPA) can bind to apoB and inhibit the lipid transfer of MTP to APOB, thereby reducing the assembly of VLDL and plasma levels of APOB-lipoprotein cholesterol [28]. Nevertheless, this is an area of burgeoning interest.

## Intracellular trafficking of VLDL

VLDLs are generated in the ER and then transported to the Golgi for secretion (Fig. 2). For precise delivery, exporting VLDLs from the ER to the Golgi may require efficient and specialized transport machinery. VLDLs are packaged into transport vesicles generated by coat complex II (COPII) to exit the ER [17]. COPII assembly on the ER surface is initiated by activating the secretion-associated Ras-related GTPase 1 (SAR1) via its guanine nucleotide exchange factor (GEF) Sect. 12 [29]. SAR1 exposes its N-terminal amphipathic α-helix upon activation and inserts into the ER membrane [30]. Activated SAR1 recruits the inner coat complex Sect. 23/Sect. 24 [31] and, subsequently, the outer coat complex Sect. 13/Sect. 31 to form COPII-coated vesicles [31]. The size of a typical COPII vesicle ranges from 60 to 70 nm in diameter [32], while lipid-containing APOB may be oversized for regular COPII vesicles [33]. To accommodate this unique cargo, specialized factors may be needed to facilitate the ER export of VLDLs [34].

Notably, germline mutations in human SAR1B, one of the two SAR1 paralogs, cause chylomicron retention disease (CMRD), an inborn metabolic defect in fat absorption due to the retention of chylomicrons (the APOB-containing lipoprotein produced in the gut) in the small intestine [35]. Consistently, selective inactivation of murine *Sar1b* in the liver depletes fasting plasma lipids due to blockage of VLDL secretion [36]. To bridge the lipid-bearing APOB particles in the ER lumen to the cytosolic COPII machinery, Surfeit 4 (SURF4) acts as the cargo receptor to selectively escort the ER-Golgi transport of lipoproteins in a physiological setting [36]. Genetic ablation of hepatic *Surf4* in mice depletes plasma lipids near zero, resulting from selective retention of lipoproteins in the ER [36]. Importantly, a genome-wide analysis (GWAS) showed a strong association between plasma LDL levels and SURF4 in humans, further supporting the essential role of the specialized SURF4-mediated lipoprotein export pathway in systemic lipid homeostasis. In addition to the profound impacts of SAR1B and SURF4 on VLDL secretion, additional factors, including transport and Golgi organization protein 1 (TANGO1), TANGO1-like (TALI) [37] and meningioma-expressed antigen 6 (Mea6) [34], are involved in the secretion of APOB-containing lipoproteins; however, how these auxiliary factors coordinate with each other has not been fully elucidated. Comprehensive reviews are cited for further reading on the biogenesis and intracellular trafficking of VLDL [38].

## Metabolism of VLDL in circulation

**Fig. 3:**
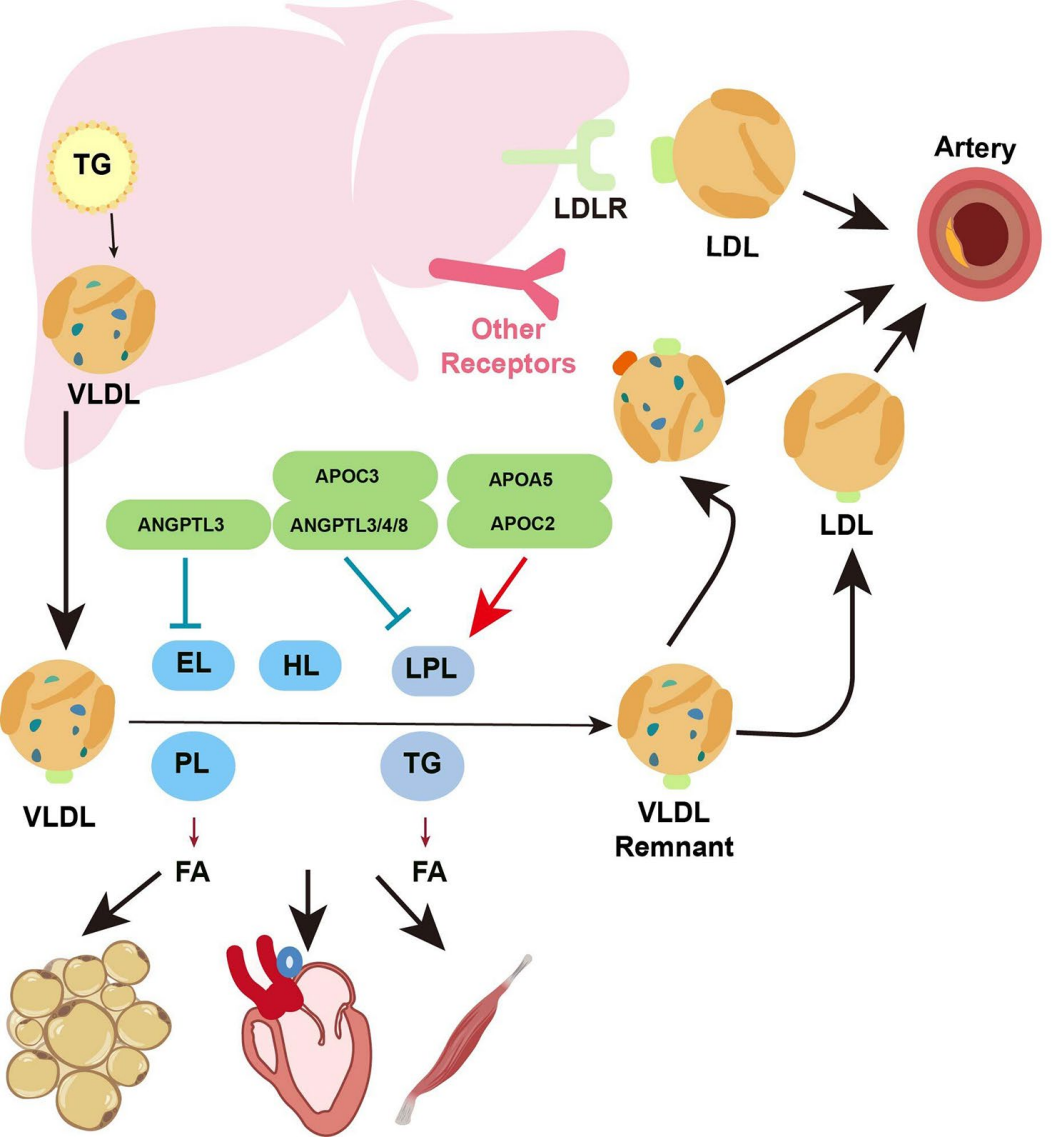
Metabolism of VLDL in circulation. After entering the bloodstream, VLDL undergoes hydrolysis by lipoprotein lipase (LPL), endothelial lipase, and hepatic lipase, leading to the release of free fatty acids from the core triglycerides and surface phospholipids. The activity of LPL is inhibited by apolipoprotein C3 (APOC3) and angiopoietin-like protein (ANGPTL)3/4/8, while it is activated by APOA5 and APOC2. When a large amount of triglycerides in VLDL is hydrolyzed, VLDL can be further metabolized into VLDL remnants, nearly half of which are absorbed by the liver, while the remaining VLDL remnants are further metabolized into low-density lipoprotein (LDL). LDL can bind to LDL receptors (LDLRs) and be taken up by the liver. Excessive VLDL remnants and LDL can deposit in the blood vessel walls, forming atherosclerotic plaques

Despite extensive research efforts, the metabolism of VLDL in the bloodstream has not been fully elucidated (Fig. 3). Once released into circulation by the liver, VLDL triglycerides and phospholipids become susceptible to hydrolysis by various lipase families, resulting in the liberation of free fatty acids. These free fatty acids can be taken up by energy-demanding cells such as those of the heart and skeletal muscle or stored in adipose tissue. Additionally, the triglycerides on VLDL can be exchanged with cholesterol esters on HDL through the action of cholesteryl ester transfer protein (CETP) [39]. Three crucial regulators of VLDL metabolism include lipase families: LPL, hepatic lipase, and endothelial lipase. As synthesized and secreted by the liver, hepatic lipase is anchored by sulfate proteoglycans (HSPGs) on the cell surface of hepatocytes and endothelial cells. Endothelial lipase is predominantly present in vascular endothelial cells of organs such as the liver, lung, kidney, and placenta [40]. LPL synthesis occurs in the parenchymal cells of white adipose tissue and energy-consuming tissues (e.g., heart, skeletal muscle, and brown adipose tissue). It is then transported to the luminal surface of vascular endothelial cells via glycosylphosphatidylinositol-anchored high-density lipoprotein-binding protein 1 (GPIHBP1). Hepatic lipase catalyzes the hydrolysis of TG and phospholipids [41], while LPL, the rate-limiting enzyme, primarily catalyzes TG [42]. Both hepatic lipase and endothelial lipase exhibit a strong affinity for phospholipids [41, 43]. Variants in LPL, hepatic lipase, and endothelial lipase have been linked to lipid traits in genome-wide association studies (GWASs) [44], underscoring their significant role in lipid metabolism. The activities of LPL, hepatic lipase, and endothelial lipase are regulated by various factors. Glycosylphosphatidylinositol anchored high-density lipoprotein binding protein 1 (GPIHBP1), APOC2, and APOA5 promote the activity of LPL, while its activity can be inhibited by members of the angiopoietin-like (ANGPTL) protein families (ANGPTL3, ANGPTL4, and ANGPTL8) and by APOC3 [42]. The inhibitory effect of ANGPTL3 on LPL is particularly pronounced when ANGPTL3 forms a complex with ANGPTL8, which is activated upon refeeding [45–47]. ANGPTL3 can also inhibit the activity of endothelial lipase [45]. Genetic cohort studies have paved the way for developing and clinically applying inhibitors targeting APOC3 and ANGPTL3, which have shown promising potential in reducing lipid levels.

After LPL, a significant portion of triglycerides are removed from VLDL, causing a change in its composition and the transformation of triglycerides into a VLDL remnant. Approximately half of the VLDL remnants are recognized and taken up by liver cells through receptors such as LDLR, LDLR-related protein (LRP), the VLDL receptor, and heparan sulfate proteoglycan (HSPG) receptors (syndecan-1) [48, 49]. The remaining 50% of VLDL remnants lacking ApoE are LDL, which is internalized by cells via receptor-mediated endocytosis through LDLR [23, 50]. LDL cholesterol and other lipids can be stored or utilized by cells. The phospholipids found in VLDL can be hydrolyzed by hepatic lipase [51] and endothelial lipase [43]. However, the specific biological function of these phospholipids on the surface of VLDL is not yet fully understood. When there is an excess of APOB-containing lipoprotein particles in the bloodstream, including VLDL, VLDL remnants, and LDL, they can deposit on the vascular wall and contribute to the development of atherosclerosis [52].

## Therapies for the reduction of LDL-C and its challenge

Numerous clinical studies have established that cholesterol, particularly LDL-C, is a causative risk factor for ASCVD. Over the past few decades, significant advancements have been made in lipid-lowering therapies. The primary lipid-lowering drugs currently used include statins, ezetimibe, and proprotein convertase subtilisin/kexin type 9 (PCSK9)-inhibiting monoclonal antibodies. Statins are the most frequently prescribed lipid-lowering medications due to their effectiveness in reducing LDL-C [53, 54]. Statins exhibit a modest triglyceride-lowering effect of only approximately 9–31% [55], depending on baseline triglyceride levels. Although PCSK9 inhibitors effectively reduce LDL-C levels, they have a limited impact on TG levels, typically reducing LDL-C levels by only approximately 10–20% [56, 57]. A reduction of 1 mmol/L (38.7 mg/dL) in both statins and nonstatin drugs has been shown to be associated with a significant 21% decrease in the risk of major cardiovascular events [58]. In recent years, mounting evidence has suggested that an elevated TRL may contribute to this residual cardiovascular risk [5]. In addition to TRLs, the enduring risk of ASCVD may be linked to elevated levels of lipoprotein (a) (Lp(a)) and chronic inflammation [59]. Lp(a) has been identified as a contributing factor to cardiovascular risk, as substantiated by both epidemiological and genetic research [60]. Ongoing clinical trials seek to validate whether diminishing Lp(a) levels can effectively reduce cardiovascular events, providing us with additional insights. Various anti-inflammatory medications have been used in individuals with ASCVD or those at risk. Notably, both canakinumab [61] and colchicines [62] have been shown to reduce the risk of ASCVD, with no significant impact on plasma lipid levels. VLDL, the precursor of LDL, serves as the primary source of TG and TRL during fasting. Targeting VLDL may reduce both TG and LDL levels.

## Inhibition of the biogenesis and lipidation of VLDL to lower blood lipids

Under the action of lipoprotein lipase, VLDL TG is hydrolyzed and converted into VLDL remnants and LDL [63]. The fasting plasma LDL-C and TG concentrations partially depend on the amount of VLDL secreted by the liver. Inhibiting the secretion of hepatic VLDL lipids can effectively reduce the level of circulating lipids through three main pathways: (1) reducing the amount of lipids needed for VLDL assembly, (2) inhibiting the lipidation of VLDL in the liver, and (3) inhibiting the transport and secretion of hepatic VLDL particles.

### 1) Targeting APOB

APOB is the structural protein for VLDL, and inhibiting the synthesis of the APOB protein can significantly reduce the synthesis of hepatic VLDL. Current therapies lower plasma lipids by inhibiting APOB synthesis [64, 65]. Mipomersen, an oligonucleotide targeting hepatic APOB mRNA, showed an efficient lipid-lowering effect with a 50% reduction in APOB [64, 65]. However, due to its liver toxicity, this medicine is exclusively approved for use and administration to patients with homozygous familial hypercholesterolemia (HoFH). As expected, any treatment that inhibits the biogenesis of APOB directly may cause hepatic steatosis, which limits its usage.

### 2) Inhibition of VLDL lipidation

As discussed above, the first step of VLDL lipidation requires MTP and PLTP, while the second step of VLDL lipidation is associated with TM6SF2 and other proteins [14, 16, 17, 21, 66]. A selective inhibitor of MTP, lomitapide, has been approved by both the FDA and EMA for use in treating HoFH [67]. Although lomitapide successfully lowered LDL-C levels by 40–50%, it was also associated with hepatic steatosis and gastrointestinal side effects [67]. MTP is necessary for the assembly of VLDL in hepatocytes by facilitating the incorporation of triglycerides into VLDL [15], and inhibition of MTP decreases the efficacy of lipids (mainly TG) export by hepatocytes, hence increasing hepatic steatosis. However, a recent clinical trial showed that lomitapide caused mild to moderate hepatic steatosis without affecting hepatic stiffness after more than nine years of follow-up [68]. However, the safety of MTP inhibition still needs to be explored.

There are currently no drugs targeting the second step of VLDL lipidation. Genetic evidence has shown that TM6SF2 loss-of-function mutations are linked to decreased plasma lipids and ASCVD [21], indicating an adequate lipid-lowering capacity and cardioprotective effect. However, genetic evidence and preclinical studies confirmed that inactivation of TM6SF2 resulted in hepatic steatosis and increased aminotransferase levels [21, 69]. Mechanisms that.

Block secretion and thereby lipid flux out of the liver are likely nonviable mechanisms due to the development of steatosis.

## Targeting the intracellular trafficking of VLDL

COPII-coated transport vehicles are thought to be responsible for transporting VLDL from the ER to the Golgi [17]. Researchers have found that there are subtle mechanisms involved in ensuring the precise and selective delivery of VLDL [36]. The membrane protein SURF4 shuttles between the ER and the Golgi apparatus, which can selectively assist nascent VLDL trafficking from the ER to the Golgi [36]. Mice lacking SURF4 had a drastic decrease in plasma VLDL, although typical secretory proteins were unaffected [36]. Inactivation of SURF4 resulted in a remarkable decrease of approximately 90% in plasma LDL-C and TG levels and entirely prevented the development of atherosclerotic plaques produced by a high-cholesterol diet coupled with overexpression of PCSK9 [36]. Although liver cholesterol and TG levels were elevated in mice with complete deficiency of SURF4, significant protection against pathological dyslipidemia and atherosclerosis was observed in heterozygotes for *Surf4* knockout without any observable hepatic damage [36, 70]. These data provide additional insight into the hypothesis that targeting VLDL secretion may be an effective method for decreasing the levels of atherogenic lipids.

TANGO1, TALI [37], and Mea6 [34] were also reported to regulate the ER-to-Golgi transport of VLDL by interacting with coat proteins of COPII. Hepatocyte-specific deletion of TANGO1 or Mea6 results in a significant defect in VLDL secretion and severe fatty liver [34, 37]. Due to their severe side effects, their potential to become lipid-lowering drug targets is limited.

## Strategies targeting VLDL metabolism in circulation

VLDL is crucial for transporting TG and cholesterol from the liver to peripheral tissues through the bloodstream. The TG carried by VLDL can undergo hydrolysis, releasing free fatty acids taken up by tissues. Similarly, the cholesterol carried by VLDL is primarily utilized by tissues through the uptake of VLDL remnants. As mentioned earlier, the core triglycerides of VLDL can be hydrolyzed by lipases present in the circulation, leading to the formation of VLDL remnants. These remnants can be directly taken up by the liver or further metabolized into LDL. The activity of LPL and endothelial lipase can be inhibited by proteins such as ANGPTL3 and APOC3. Newly published research indicates that inhibiting ANGPTL3 and APOC3 can significantly reduce circulating levels of triglycerides and cholesterol [71]. Clinical trials have also shown promising results, highlighting the potential of these agents as effective lipid-lowering agents.

### 1) Targeting ANGPTL3

ANGPTL3 is an inhibitor of both LPL and endothelial lipase [45]. Genetic studies have shown that loss-of-function mutations in ANGPTL3 significantly reduce plasma TG and cholesterol levels and have significant cardiovascular protective effects [72, 73]. Multiple methods have been explored for inhibiting ANGPTL3, including monoclonal antibodies, antisense oligonucleotides (ASOs), and mRNA interference (mRNAi) [45]. Preclinical studies have shown that inactivation of ANGPTL3 can significantly decrease TG levels and promote the clearance of VLDL remnants [45, 74–76]. Clinical studies have shown that ANGPTL3 inhibitors have very good lipid-lowering effects [77, 78]. Inhibition of ANGPTL3 in combination with statins induced a 47% reduction in LDL-C and a 55% reduction in TG in patients with familial hypercholesterolemia or refractory hypercholesterolemia [77, 78]. We hypothesize that changes in VLDL metabolism may be an important mechanism underlying the lipid-lowering effect of ANGPTL3 inhibitors [79]. Studies have shown that ANGPTL3 inhibitors can lower plasma lipids through both LDLR-dependent and LDLR-independent pathways [80], but the exact underlying mechanisms are still unknown. ANGPTL3 has been shown to hasten VLDL processing through an LDLR-independent mechanism, with VLDL being removed before LDL production [80]. LDLR-independent pathways also enable the combination of this lipid-lowering strategy with LDLR-dependent lipid-lowering strategies such as statins. Evinacumab, a monoclonal antibody that inhibits ANGPTL3, has gained approval for treating HoFH in the European Union (EU), United Kingdom (UK), and the United States (US). Conversely, the development of vupanorsen, an antisense oligonucleotide (ASO) aimed at reducing hepatic ANGPTL3 production, was halted due to hepatic side effects.

### 2) Targeting ANGPTL4

ANGPTL4 is produced by various cells and tissues, such as the liver and adipose tissue, and predominantly controls LPL activity during fasting [81]. Genetic research has validated the role of ANGPTL4 in regulating plasma TG levels. Loss-of-function mutations in ANGPTL4 correlate with lower plasma TG levels and a reduced risk of ASCVD [82]. Nonetheless, developing anti-ANGPTL4 strategies is challenging because complete ANGPTL4 inactivation across the body in mice can lead to severe clinical complications. Mice without ANGPTL4 exhibit mesenteric lymphadenopathy and undergo a significant acute phase response [83].

### 3) Targeting APOC3

APOC3 is an endogenous antagonist of LPL and hepatic lipase. APOC3 is a protein present on certain lipoproteins in the body, including VLDL and LDL. Researchers have found a correlation between APOC3 LOF mutations and lower plasma triglyceride levels as well as a decreased risk of cardiovascular disease that was 40% lower [84, 85]. Recent research has focused on developing drugs that target APOC3 as potential therapies for hypertriglyceridemia and other lipid-related disorders. Studies have shown that inhibiting APOC3 can reduce triglyceride levels by 70.9% and improve lipid profiles [86–88]. ASOs, monoclonal antibodies, and small molecule inhibitors are in development to target APOC3. Volanesorsen, an ASO directed at APOC3, has gained approval in Europe for treating familial chylomicronemia syndrome (FCS) patients. Although APOC3 inhibition has shown promise in both preclinical and clinical studies, additional research is needed to comprehensively grasp its therapeutic potential and long-term safety implications. Apolipoprotein C2 (APOC2), an exchangeable small apolipoprotein on TRL, activates LPL. Notably, APOC2 mimetic peptides reduce TG levels by displacing APOC3 from TRL, alleviating the inhibitory impact of APOC2 on LPL [89].

## Review strengths and limitations

This review primarily focuses on clinical aspects and summarizes the research on the composition, production, metabolism, and other aspects of VLDL. The possibility of intervening in VLDL by regulating these aspects has also been investigated. Not only does it provide insights from a clinical perspective, but it also includes fundamental research that can benefit both clinical and basic researchers. However, our review has several limitations. Due to the constraints of article length, our description of the underlying mechanisms may not be thorough enough. Our focus is on elucidating the metabolism of VLDL in circulation and its potential targets, and reviews of its composition and generation may not be thorough enough.

## Challenge and future directions

Current clinical guidelines and research on lipid-lowering effects on reducing ASCVD risk primarily revolve around LDL, with limited attention given to VLDL metabolism and clearance. The significance of VLDL as a precursor to TRL, a major source of remnant cholesterol in the fasting state, has yet to be fully appreciated. A burgeoning body of literature reveals that VLDL-C may account for nearly half of APOB-related myocardial infarctions, piquing interest in the potential role of VLDL-C in reducing the residual risk of ASCVD [10]. Remnant cholesterol has been proposed as a new opportunity for reducing residual cardiovascular risk [6, 66], indicating the importance of targeting VLDL. Recent studies have also demonstrated that modulating the composition of VLDL through lipases such as LPL, hepatic lipase, or endothelial lipase can considerably enhance both LDLR-dependent and nondependent clearance pathways for VLDL remnants [86–88, 90]. These strategies can effectively reduce triglycerides, remnant cholesterol, and LDL-C by targeting VLDL. However, despite their potential, numerous challenges and difficulties remain. Existing approaches to inhibit VLDL secretion can lead to hepatic lipid accumulation, which may limit their future applications. Therefore, developing better targeted therapies to reduce VLDL secretion without causing hepatic lipid accumulation is crucial. Inhibiting SURF4 to decrease VLDL secretion and circulating lipids has been shown to be dose dependent and safe [36], suggesting that it has the potential to lower plasma lipids without inducing hepatic steatosis.

Furthermore, novel lipid-lowering strategies are needed to inhibit VLDL assembly and, in combination, prevent intracellular lipid excess by promoting free fatty acid oxidation or reducing triglyceride synthesis. Promising avenues reside in strategies promoting VLDL metabolism and clearance, such as ANGPTL3 inhibitors [45]. In patients with HoFH, ANGPTL3 inhibitors can lead to a nearly 50% reduction in LDL-C [77], offering hope for FH patients and improving lipid-lowering options for individuals with high residual cardiovascular risk or statin intolerance. Research has established a causal relationship between VLDL remnants and cardiovascular risk [10], highlighting the importance of targeting VLDL in lipid-lowering strategies to address the current challenges in lipid-lowering treatment. Currently, there is a need for simpler and more reliable methods for monitoring circulating VLDL in patients, which can circumvent difficulties in assessing the therapeutic efficacy of interventions targeting VLDL. Therefore, further research is needed to explore and develop accessible and cost-effective methods for detecting circulating VLDL.

## Data Availability

No data were generated or analyzed for this manuscript.

## Abbreviations

APOB: Apolipoprotein B
ANGPTL: Angiopoietin-like protein
APOA5: Apolipoprotein A5
APOC2: Apolipoprotein C2
APOE: Apolipoprotein E
ASCVDs: Atherosclerotic cardiovascular disease
ASO: Antisense oligonucleotide
CIDEB: Cell death-inducing DFF45-like effector B
CETP: Cholesteryl ester transfer protein
CMRD: Chylomicron retention disease
COPII: Coat complex II
ER: Endoplasmic reticulum
ERGIC: ER-Golgi intermediate compartment
GWAS: Genome-wide analysis study
HDL: High-density lipoprotein
HMG-CoA: 3-hydroxy-3-methylglutaryl coenzyme A
HoFH: Homozygous familial hypercholesterolemia
IDL: Intermediate-density lipoprotein
GPIHBP1: Glycosylphosphatidylinositol anchored high-density lipoprotein binding protein 1
LDL: Low-density lipoprotein
LPCAT3: Lysophosphatidylcholine acyltransferase 3
LPL: Lipoprotein lipase
LRP: LDLR-related protein
Mea6: Meningioma-expressed antigen 6
MTP: Microsomal triglyceride transfer protein
PCSK9: Proprotein convertase subtilisin/kexin type 9
PLTP: Phospholipid transfer protein
TMEM41B: Transmembrane protein 41B
RER: Rough endoplasmic reticulum
SAR1: Secretion-associated Ras-related GTPase 1
VLDL: Very low-density lipoprotein
TALI: TANGO1-like
TANGO1: Transport and Golgi organization protein 1
TG: Triglyceride
TM6SF2: Transmembrane 6 superfamily member 2
TRL: Triglyceride-rich lipoprotein
